# Lupus nephritis is associated with poor pregnancy outcomes in pregnant SLE patients in Cape Town: a retrospective analysis

**DOI:** 10.11604/pamj.2015.22.365.7897

**Published:** 2015-12-14

**Authors:** Lindisa Mbuli, Darlington Mapiye, Ikechi Okpechi

**Affiliations:** 1 Department of Medicine, Groote Schuur Hospital and University of Cape Town, Cape Town, South Africa; 2 South Africa National Bioinformatics Institute (SANBI), University of the Western Cape, Cape Town, South Africa; 3 Division of Nephrology and Hypertension, University of Cape Town, Cape Town, South Africa

**Keywords:** Lupus, Africans, pregnancy, maternal, foetal, neonatal, pre-eclampsia, SLE

## Abstract

**Introduction:**

Systemic lupus erythematosus (SLE) is a multi-system auto-immune disease common in females of child-bearing age. The effect of pregnancy on SLE and vice versa have not been well characterised in Africans. The aim of this study is to describe the pregnancy outcomes of patients with SLE presenting to the maternity department of Groote Schuur Hospital, Cape Town.

**Methods:**

This study was designed as a retrospective review of records of pregnant women known with SLE and followed-up at the maternity section of Groote Schuur Hospital. The duration of survey was from the 1^st^ January 2003 to 31^st^ December 2013.

**Results:**

There were 61 pregnancies reviewed in 49 patients; 80.3% of the pregnancies were in patients of mixed ancestry and the rest (19.7%) in black African patients. The mean age at presentation of the current pregnancy was 27.2±5.0 years. Mean gestational age at presentation and delivery was 13.0 ± 6.0 weeks and 28.9 ± 9.8 weeks respectively and 47.5% of the pregnancies were in patients with lupus nephritis (LN). Thirty nine (63.9%) pregnancies reached the third trimester and 11.5% of all pregnancies ended in the first trimester. There was a lower number of live births to mothers of African ancestry than to those of mixed ancestry (p=0.001). In 55.7% of the pregnancies, no flare was reported while a renal flare was reported in 23%. Pregnancies in patients with LN had higher frequencies of flares (58.6% vs 31.3%; p=0.032), pre-eclampsia (34.5% vs 12.5%; p=0.041), longer stay in hospital (12.0 ± 9.1 days vs 6.1 ± 5.1 days; p=0.004) and low birth weight babies (1.94 ± 1.02 kg vs 2.55±0.95 kg; p=0.046) than in patients without LN. Only 36 (59%) of the neonates were discharged home alive and of these 2 (5.6%) were to mothers of black African ancestry (p=0.001).

**Conclusion:**

Increased lupus activity in pregnant SLE patients may account for the increased deaths of neonates born to SLE mothers. Patients of black African descent and those with LN tend to have a poorer outcome. A multi-disciplinary approach to the management of SLE patients (of child-bearing age or pregnant) needs to be further assessed for better outcomes.

## Introduction

Systemic lupus erythematosus (SLE) is an autoimmune disease with a manifestation of complex interplay between genetic factors, hormones, autoantibodies and environmental factors [1]. Like most autoimmune diseases it affects mostly women of childbearing age although all ageand ethnic groups are susceptible [2, 3]. The evolution of SLE is known to be changed by natural hormonal events (e.g. menstrual period, menopause and pregnancy). The care of pregnant women with SLE as well as pregnancy outcomes in women with SLE has been reported to have significantly improved even though these reports are often from developed countries [4]. Pregnancy in SLE is, however still considered “high-risk” especially in low-income countries where all the facilities or options of therapy may not be available. Adverse short term and long term maternal outcomes that have been reported in SLE includes maternal death, stroke, hypertension, pre-eclampsia or eclampsia and LN with accelerated end organ damage requiring dialysis [5]. In one study, SLE patients were twice more likely to have a caesarean section and their neonates were more likely to be of low birth weight, preterm delivery and had higher frequencies of congenital malformations than reference patients [6]. Data is limited from countries in Africa, although one study has previously reported no loss to maternal life, 13% occurrence of mild flares, 33.3% pre-eclampsia, 77% live births and 14% intra-uterine growth retardation [7]. We therefore sought to retrospectively assess the impact of pregnancy on SLE and vice versa at Groote Schuur Hospital, Cape Town and to identify, where possible, factors associated with the outcome.

## Methods

This study was approved by the University of Cape Town Human Research Ethics Committee (HREC REF 038/2013). Records of patients known with SLE meeting the American College of Rheumatology criteria for SLE 1982 (revised in 1997) who presented to the GSH gynaecology emergency unit, ante-natal clinic or maternity wards in gestation from 1 January 2003 to 31December 2013 were accessed for retrospective analysis. The patients’ records were identified using the attendance registers in the relevant departments (Rheumatology and Nephrology) involved in the care of patients with SLE. Patients were excluded if they didn't meet the American College of Rheumatology criteria for SLE, if they had mixed connective tissue disease of those who presented to the Obstetrics department outside the period of the study. Records obtained included ethnicity, age of the patient at presentation, gestational age at presentation, history of chronic diseases (hypertension, diabetes) and current and past gynaecological and obstetric history. Complications that occurred during pregnancy such as pre-eclampsia, flare of SLE (defined as a change in clinical and/or serological parameters requiring adjustment of doses of immunosuppression were categorised as renal, skin, joint, or any combination of these) [8], pre-term delivery, and method of delivery were documented. Pre-eclampsia was as diagnosed by the attending obstetrician. Duration of maternal hospitalisation and gestational age at delivery were also recorded. Foetal outcomes were documented as birth weight (normal or low birth weight), duration of hospitalisation after delivery and status of the neonate at discharge (alive or died). Various terms used were defined following South African standards [9]: Birth weight and gestational age: Low birth weight: neonatal weight 1000 - 2499g; Pre-term baby: birth at gestational age of 28-37 weeks; Term baby: birth at gestational age of > 37 weeks; Pregnancy outcomes: Miscarriage: pregnancy loss from time of conception until 24 weeks and 28 weeks of gestation or delivery of foetus below 500g in weight or; Pre-term labour: the onset of labour before 37 completed gestational weeks; Term labour: the onset of labour after 37 completed gestational weeks; a stillbirth: is a baby born dead.

### Statistical Analysis

The data were analysed using IBM SPSS Statistics 21 software (SPSS, Chicago, IL). Categorical variables were presented as percentages and continuous variables as means ± SD. Comparison was made between pregnancies in patients with LN and those without LN using the Student's t-test, chi-square test or Fisher's exact test. Significant P-value was taken as P < 0.05.

## Results

### Baseline demographics features of patients

**Figure 1:**
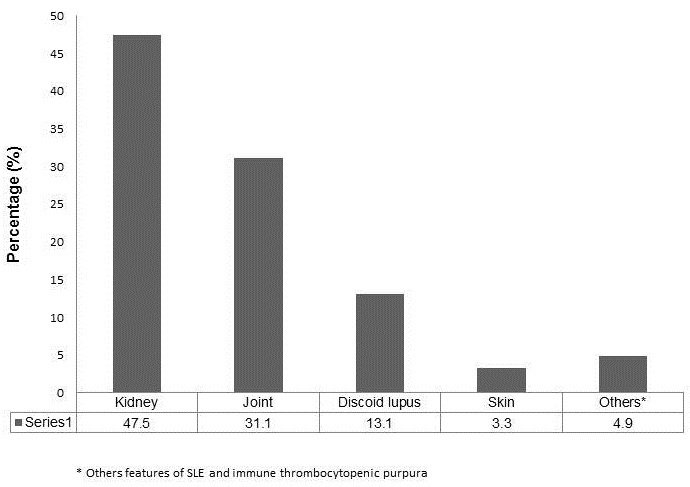
Main clinical manifestation of SLE in all the pregnancies assessed

Initially, we reviewed the data of 62 pregnancies in 50 women. One patient was excluded as there was inadequate evidence to show that she fulfilled the ACR criteria for the diagnosis of SLE. Hence, the baseline features of 61 singleton pregnancies (49 women) that fulfilled the ACR classification criteria for a diagnosis of SLE are shown in Table 1, and 12 women had more than pregnancy during the course of the study. The mean age at presentation (for the assessed pregnancy) was 27.2 ± 5.0 years, most of the patients (80.3%) were of mixed ancestry and 47.5% of the pregnancies occurred in patients known with biopsy proven LN (Figure 1). Treatment records at presentation included use of glucocorticoids (67.2%), cyclosporine A (9.8%), Azathioprine (23.0%), chloroquine (50.8%) and various anti-hypertensive agents (none - 50.8%, 1 anti-hypertensive agent - 23.0%, 2 or more anti-hypertensive agents - 26.2%) (not shown in Table).

**Table 1:** Baseline demographics features of SLE pregnancies

Variable	Frequency (n=61)*
Age at SLE diagnosis (Years)	23.3 ± 6.3
Age at presentation - current pregnancy (years)	27.2 ± 5.0
Race:	
Black Africans	12 (19.7%)
Mixed ancestry	49 (80.3%)
GA at presentation (weeks)	13.0 ± 6.0
Trimester at presentation	
First	41 (67.2%)
Second	17 (27.9%)
Third	3 (4.9%)
Kidney involvement in SLE:	
Lupus nephritis	29 (47.5%)
No-lupus nephritis	32 (52.5%)
History previous pregnancies (%):	
Primigravida	20 (32.8%)
Others	41 (67.2%)

* This number represents the total number of pregnancies assessed in this study

### Maternal and foetal features and outcomes

**Figure 2:**
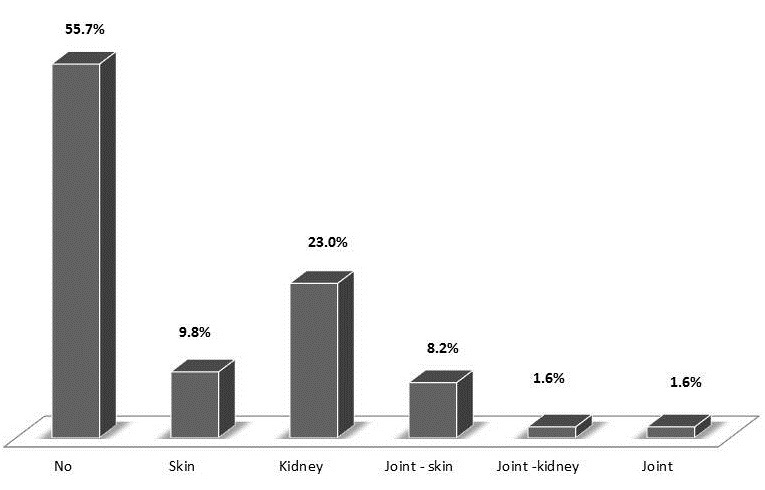
Organ involvement during flares in SLE pregnant patients

**Figure 3:**
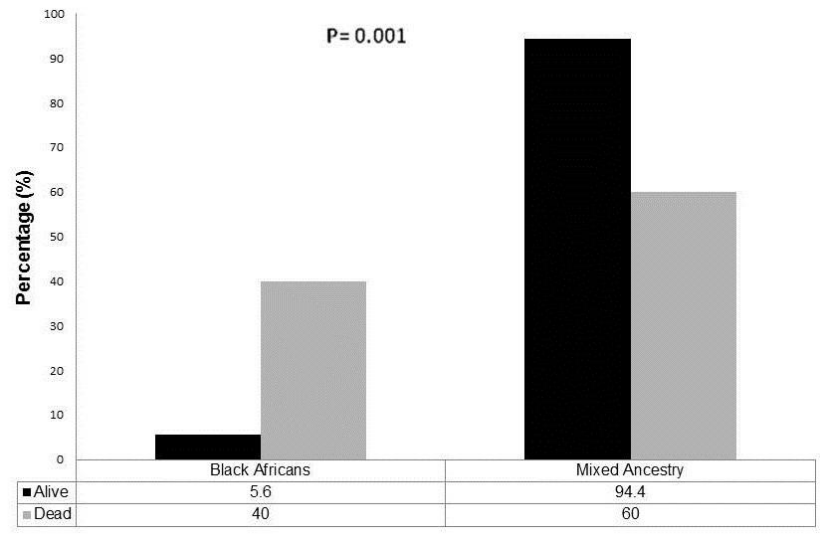
Neonatal discharge outcomes by maternal race

Table 2,Table 3 summarises the recorded maternal and foetal features and outcomes from pregnancy to time of delivery. The mean gestational age at delivery was 28.9 ± 9.8 weeks, most of the deliveries (63.9%) occurred in the third trimester of pregnancy and over half of deliveries (52.5%) were either by evacuation of retained products of conception or Caesarean section. Pre-eclampsia occurred in 23% of all the pregnancies. A lupus flare was documented in 44.3% of pregnancies; most flares predominantly affected the kidneys (23.0%) (Figure 2). At birth, 64% of neonates were born alive, 9.8% were stillbirths (Table 3). However, only 59% of the neonates were discharged home alive as three infants died shortly after birth. The average birth weight of infants was 2.24 ± 1.02 kg and 54.5% were low birth weights. The average duration of hospitalisation post-delivery for neonates was 9.2 days (min - 0; max - 70 days). Neonates born to black African mothers significantly had lower survival rates than those born to mothers of mixed ancestry (5.6% vs 94.4%; p=0.001) (Figure 3).

**Table 2:** Maternal features and outcomes (n=61)*

Variable	Value
Mean gestational age at delivery (weeks)	28.9 ± 9.8
Trimester at time of delivery (%)	
First Trimester	11.5
Second Trimester	24.6
Third Trimester	63.9
Method of delivery (%)	
Normal vaginal delivery	47.5
Evacuation**	24.6
Caesarean section	27.9
Pre-eclampsia (%)	23.0
Previous pregnancy loss #	36.1

* This number represents the total number of pregnancies assessed in this study

** This refers to evacuation of the retained products of conception

# Represents all previous termination of pregnancy, stillbirths and miscarriages

**Table 3:** Foetal features and outcomes (n=61)*

Variable	Value
Average birth weight (kg)	2.24 ± 1.2
Birth weight (%)	
Low birth weight	54.5%
Normal birth weight	45.5%
Mean duration of Hospitalization (days)	9.2 (0-70) [min –max]
Status at birth (%)	
Alive	64.0
Medical termination	3.3
Miscarriage	23.0
Stillbirth	9.8
Outcome at discharge (%)	
Alive	59.0
Died	41.0

* The number represents the total number of deliveries assessed in this study

### Differences in features and outcomes in pregnancies of patients with lupus nephritis and those without kidney involvement

Table 4 shows the differences in pregnancy features and outcomes between patients with LN and those without LN. Importantly, pregnant women with LN had a higher frequency of flares (58.6% vs 31.3%; P=0.032), higher rate of pre-eclampsia (34.5% vs 12.5%, P=0.041), longer duration of hospitalization (12.0 ± 9.1 vs 6.1 ± 5.1 days; P=0.004) and lower mean birth weight of the neonate (1.94 ± 1.02 vs 2.55 ± 0.95 kg; P=0.046) than those without kidney involvement. Patients with LN had a lower neonatal survival (51.7%) at discharge compared to those without LN (65.6%), however, this was not significantly different (p=0.270) (Table 3).

**Table 4:** Differences in pregnancy outcomes between patients with lupus nephritis and patients with SLE without nephritis

Variable	Lupus Nephritis (n=29)	SLE without nephritis (n=32)	P-value
Mean gestational age at delivery (weeks)	28.0 ± 9.0	29.6 ± 10.6	0.532
Flare (%)	58.6	31.3	**0.032**
Pre-eclampsia (%)	34.5	12.5	**0.041**
Mean Maternal Hospital stay (days)	12.0 ± 9.1	6.1 ± 5.1	**0.004**
Pregnancy outcomes (%)			0.218
Miscarriage	20.7	25.0	
Medical termination	6.9	0	
Still birth	10.3	9.4	
Preterm live birth	44.8	28.1	
Term live birth	17.2	37.5	
Mode of delivery (%)			0.427
Evacuation**	24.1	25.0	
Normal vaginal delivery	55.2	40.6	
Caesarean section	20.7	34.4	
Neonatal outcomes			
Alive on discharge (%)	51.7	65.6	0.270
Mean birth weight (kg)	1.94 ± 1.02	2.55 ± 0.95	**0.046**
Mean neonatal hospitalisation (days) [Min-Max]	9.13 [1 – 70]	9.24 [0 – 52]	0.984

** This refers to evacuation of retained products of conception

## Discussion

Systemic lupus erythematosus is predominantly a disease of women of child-bearing age; it is therefore not uncommon or unexpected that these patients may present with pregnancy and this can be associated with hormonal changes likely to increase activity of lupus and affect the outcome of pregnancy. The key findings in the current study relates to our observation of overall increased neonatal morbidity and mortality especially if born to patients known with LN or patients of black African ancestry. We also noted a relatively high neonatal mortality in comparison with the results of studies published elsewhere (Table 5) [7, 10–15]. We consider the high neonatal mortality in our study as a reflection of the severity of lupus activity and not of the state of healthcare in South Africa as all our patients were treated in a large tertiary health care facility. Increased lupus activity [13], previous obstetric history (i.e. previous pregnancy loss) [16], renal involvement in lupus [17] and secondary anti-phospholipid syndrome [18] have been identified as predictors of pregnancy loss in patients with lupus and some of these factors might explain the poor outcome in our study. In one study, Yang et al assessing lupus activity with the SLEDAI score reported the incidence of preeclampsia/eclampsia, foetal loss, and preterm birth to be significantly higher in patients with active pregnancy related lupus compared to those with stable pregnancy related lupus (p < 0.05) [19]. They also reported that despite receiving a more vigorous glucocorticoid treatment, mothers with active pregnancy related lupus had a poorer prognosis compared to those with stable lupus in pregnancy (p < 0.001) [19]. Although lupus activity scores were not directly assessed in our study, activity was indirectly measured through the occurrence of lupus flares which we found to be more common in those with LN (Table 4). Also, we observed that in 36.1% of mothers, there was a history of pregnancy loss in previous pregnancies and mothers with such history had a higher chance or recurrent loss of current pregnancy (results not shown). Ramsey-Goldman et al have shown in their study of SLE patients with either positive or negative anticardiolipin antibodies that in both groups, if there was an adverse outcome in their first pregnancy had at least a 50% chance of another adverse outcome in their second pregnancy [16]. Hence, SLE patients with previous pregnancy loss may be at greater risk of loss of conception in subsequent pregnancies. Although we did not find any significant correlation between previous pregnancy loss and outcome in current pregnancy, this may be an important factor to consider in patients with SLE who become pregnant. We also found that LN was associated with increased flare in pregnancy (p=0.032), increased frequency of pre-eclampsia (p=0.041), low neonatal birth weight (p=0.046) and increased maternal duration of hospitalization all of which are surrogates of poor maternal and foetal outcomes (Table 4). One meta-analysis of pregnancy outcomes in patients with SLE and LN comprising of 1842 patients with 2751 pregnancies reported the rates of lupus flare, pre-eclampsia, spontaneous abortion, stillbirth, and neonatal deaths to be 25.6%, 7.6%, 16%, 3.6%, and 2.5% respectively [11]. However, in a multicentre study of 81 women (113 pregnancies) with pre-existing biopsy-proven LN that assessed the risk factors for complicated pregnancy (foetal loss and renal flares), Imbasciati et al reported nine spontaneous abortions, one stillbirth and five neonatal deaths [20]. They also reported that renal flares during and after pregnancy are not uncommon and can be predicted by renal status assessed before pregnancy. Taken together, these findings show that in patients with SLE and LN there is an increased risk of maternal and foetal complications and further supports the need for timing of pregnancy relative to SLE activity and multispecialty care of these patients.

**Table 5:** Pregnancy outcomes reported from various studies worldwide

Country	Author (Year) [Ref]	Number of pregnancies	Pregnancy outcomes		
			Miscarriage (%)	Premature deliveries (%)	Live Birth (%)
China	Liu et al. (2012) [10]	111	22.5	25.2	74.8
Systemic review & meta-analysis	Smyth et al. (2010) [11]	2751	16 **	39.4	74.5
Portugal	Carvalheiras et al. (2010)[12]	51	6	16	74
USA	Clowse et al.(2005) [13]	267	7	40	85.8
South Africa	Whitelaw et al. (2008) [7]	47	21	39	77
India	Chandran et al. (2004) [14]	52	28.6	1.9	46.1
Taiwan	Wong et al (2006) [15]	24	8.3	33.3	91.6
This study (South Africa)	Mbuli et al (2015)	61	23	36.1	64.0

** Excluded 5.9% induced abortions

Finally, we report from our study lower neonatal survival for those of black African ancestry compared to those of mixed ancestry (p=0.001) (Figure 3). As all the mothers and newly born would have received the same level of care from our hospital, this outcome is thought to be related to an increased severity of lupus in those of African ethnicity or may be due to differences in other socio-demographic factors such as level of education, nutritional status and attitude relating to the use of medical facilities (the later factors were not assessed in this study). In this study, among patients of African ancestry, 58.3% had a flare during pregnancy compared to 40.8% in those of mixed ancestry. SLE has been reported to be more severe in those of African descent often with less favourable outcomes than in Caucasians and in those of poor socio-economic status [21–24]. In the LUMINA study, 34 of 288 SLE patients had died within 5 years of study onset; most were African Americans and some factors that were associated with mortality included poverty, less than full-time employment, difficulty in accessing health care and cardiovascular and renal involvement [23]. Ward et al also reports on univariate analyses of their study that mortality rates increased with age and were higher among males, blacks, those without private medical insurance, and those living in census tracts with lower household incomes in the US [24]. Other factors relevant to the ethnic variance in the outcome may be genes related to progression of renal disease, such as ACE polymorphisms. These findings support the role of race and socio-demographic factors in the outcome of patients with SLE and this can be extended to the outcomes observed in pregnancy as in our study. The higher severity of lupus with increased activity during pregnancy in black African patients may suggest that such patients will require closer monitoring of lupus flares and perhaps a more intensive follow-up at the ante-natal clinics in order to improve outcome of pregnancy. Our results are limited in some ways including the retrospective design of this study which meant that not all patients’ records could have been obtained. For example the results of serological assays for SLE (anti-double stranded DNA antibodies, anti-SSA/Ro and anti-SSB/La antibodies, antiphospholipid antibodies, C3 and C4 compliment level) were not always available and hence not presented in our data. Also, the retrospective design means that the interpretation of flares (disease activity) was sometimes done retrospectively as the attending physicians made the diagnosis of flares differently and the small sample size of this study may be a limitation of how the results are interpreted; although other published studies have reported on similar sample sizes.

## Conclusion

Our results suggest that pregnancy in patients with SLE is associated with a relatively poor maternal and foetal outcome, particularly in those with LN and those of African descent. There is need for intensive monitoring of these patients during pregnancy and perhaps a multi-disciplinary approach to the management of SLE patients, including an obstetrician (optimally a maternal foetal medicine specialist), a rheumatologist, and a nephrologist. Preconception counselling in non-pregnant SLE patients to increase awareness of the disease should be routinely carried where the goal is to optimise her health before becoming pregnant. Such an approach could help in reducing the relatively high maternal and foetal morbidity and mortality often reported in developing countries like South Africa.

## References

[1] Tiffin N, Adeyemo A, Okpechi I (2013). A diverse array of genetic factors contribute to the pathogenesis of systemic lupus erythematosus. Orphanet J Rare Dis..

[2] Siegel M, Stanley L (1973). The epidemiology of systemic lupus erythematosus. Semin Arthritis Rheum..

[3] Danchenko N, Satia JA, Anthony MS (2006). Epidemiology of systemic lupus erythematosus: a comparison of worldwide disease burden. Lupus..

[4] Lateef A, Petri M (2013). Managing lupus patients during pregnancy. Best Pract Res ClinRheumatol..

[5] Clowse MEB (2007). Lupus Activity in Pregnancy. Rheum Dis Clin North Am..

[6] Wallenius M, Salvesen KÅ, Daltveit AK, Skomsvoll JF (2014). Systemic lupus erythematosus and outcomes in first and subsequent births based on data from a national birth registry. Arthritis Care Res (Hoboken)..

[7] Whitelaw D, Hall D, Kotze T (2008). Pregnancy in systemic lupus erythematosus: a retrospective study from a developing community. ClinRheumatol..

[8] Gordon C, Jayne D, Pusey C, Adu D, Amoura Z, Aringer M (2009). European consensus statement on the terminology used in the management of lupus glomerulonephritis. Lupus..

[9] Cronje HS, Cilliers JB, Pretorius MS (2011). Clinical Obstetrics. A South African perspective.

[10] Liu J, Zhao Y, Song Y, Zhang W, Bian X, Yang J (2012). Pregnancy in women with systemic lupus erythematosus: a retrospective study of 111 pregnancies in Chinese women. J MaternFetal Neonatal Med..

[11] Smyth A, Oliveira GH, Lahr BD (2010). A systematic review and meta-Analysis of pregnancy outcomes in patients with systemic lupus erythematosus and lupus nephritis. Clin J of Am SocNephrol..

[12] Carvalheiras G, Vita P, Marta S, Trovão R, Farinha F, Braga J (2010). Pregnancy and systemic lupus erythematosus: review of clinical features and outcome of 51 pregnancies at a single institution. Clin Rev Allergy Immunol..

[13] Clowse ME, Magder LS, Witter F, Petri M (2005). The impact of increased lupus activity on obstetric outcomes. Arthritis Rheum..

[14] Chandran V, Aggarwal A, Misra R (2005). Active disease during pregnancy is associated with poor foetal outcome in Indian patients with systemic lupus erythematosus. RheumatolInt..

[15] Wong C, Chen T, Lee C, Lin C, Chen C (2006). Outcome of pregnancy in patients with systemic lupus erythematosus. Taiwan J Obstet and Gynecol..

[16] Ramsey-Goldman R, Kutzer JE, Kuller LH (1992). Previous pregnancy outcome is an important determinant of subsequent pregnancy outcome in women with systemic lupus erythematosus. Am J of Reprod Immunol..

[17] Witter FR (2007). Management of the high-risk lupus pregnant patient. Rheum Dis Clin North Am..

[18] Clowse ME, Magder LS, Witter F, Petri M (2006). Early risk factors for pregnancy loss in lupus. Obstet Gynecol..

[19] Yang H, Liu H, Xu D (2014). Pregnancy-related systemic lupus erythematosus: clinical features, outcome and risk factors of disease flares-a case control study. PloS one..

[20] Imbasciati E, Tincani A, Gregorini G (2009). Pregnancy in women with pre-existing lupus nephritis: predictors of fetal and maternal outcome. Nephrol Dial Transplant..

[21] Reveille JD, Bartolucci A, Alarcón GS (1990). Prognosis in systemic lupus erythematosus. Arthritis Rheum..

[22] Mody GM, Parag KB, Nathoo BC, Pudifin DJ, Duursma J, Seedat YK (1994). High mortality with systemic lupus erythematosus in hospitalised African blacks. Br J Rheumatol..

[23] Alarcón GS, McGwin G, Bastian HM (2001). Systemic lupus erythematosus in three ethnic groups: VIII. Predictors of early mortality in the LUMINA cohort. Arthritis Rheum..

[24] Ward MM, Pyun E, Studenski S (1995). Long-term survival in systemic lupus erythematosus:patient characteristics associated with poorer outcomes. Arthritis Rheum..

